# A Distinct Difference Between Air and Mucosal Temperatures in Human Respiratory Tract

**DOI:** 10.3389/fmed.2021.650637

**Published:** 2021-07-30

**Authors:** Mehdi Khosravi, Ruei-Lung Lin, Ashish P. Maskey, Subodh Pandey, An-Hsuan Lin, Lu-Yuan Lee

**Affiliations:** ^1^Department of Medicine, University of Kentucky Medical Center, Lexington, KY, United States; ^2^Department of Physiology, University of Kentucky Medical Center, Lexington, KY, United States

**Keywords:** airway, lung, mucosa, TRPV1, exhaled breath, inflammation, bronchoscopy

## Abstract

Extensive evidence indicates that several types of temperature-sensitive ion channels are abundantly expressed in the sensory nerves innervating airway mucosa. Indeed, airway temperature is known to play an important role in regulating respiratory functions. However, the actual airway mucosal temperature and its dynamic changes during the respiratory cycle have not been directly measured. In previous studies, airway tissue temperature was often estimated by indirect measurement of the peak exhaled breath temperature (PEBT). In view of the poor thermal conductivity of air, we believe that the airway tissue temperature cannot be accurately determined by the exhaled air temperature, and this study aimed to test this hypothesis. We applied a miniature rapid-response temperature probe to measure directly the mucosal temperatures of trachea, major, lobar, and segmental bronchi in eight human subjects during a bronchoscopy procedure. Unlike the air temperature in the airway lumen, the mucosal temperature in these airway segments remained relatively stable and did not exhibit the phasic changes synchronous with respiratory cycles. The airway mucosal temperature increased progressively from the extra-thoracic trachea (35.7 ± 0.2°C) toward the segmental bronchus (36.9 ± 0.2°C). Most importantly, the temperatures measured directly at the mucosa of all these airway segments were substantially higher than the PEBT (31.7 ± 0.8°C). The recent findings of a close association between an increased PEBT and airway tissue inflammation have revealed the implication and potential of incorporating the PEBT measurement in the future clinical diagnosis of airway inflammation. Therefore, it is imperative to recognize this distinct difference in temperature between airway mucosa and exhaled air.

## Introduction

A change in airway temperature can cause profound changes in airway functions under both physiological and pathophysiological conditions. For example, a significant drop in airway mucosal temperature during exercise or breathing cold dry air is known to be responsible for triggering the exercise-induced bronchoconstriction in asthmatics (1, 2). Conversely, an increase in exhaled air temperature associated with inflammation (e.g., during acute asthma exacerbation) has been reported in both adults and children (3, 4). Furthermore, an increase in airway temperature induced by hyperventilation with warm humidified air triggered airway hypersensitivity and bronchoconstriction in anesthetized rats (5, 6). Indeed, specific “temperature sensors” such as transient receptor potential (TRP) receptors are abundantly expressed in vagal sensory nerves innervating the mucosa of the entire respiratory tract in humans and various animal species (7–11). TRPs are ligand-gated non-selective cation channels, and many of them are extremely sensitive to changes in temperature within the physiological range (8, 12). More importantly, activation of these TRP-expressing airway nerves is believed to be involved in the manifestation of various symptoms (e.g., cough, airway irritation, bronchoconstriction, etc.) found in patients with acute or chronic airway inflammatory diseases (10, 13, 14). However, the data of mucosal temperature recorded by a direct measurement in human airways is lacking because the procedure is difficult to perform in awake subjects. As such, in those previous studies the tissue temperature of intrathoracic airways was estimated by a measurement of the peak (end-tidal) exhaled breath temperature [PEBT; (3, 4, 15–17)]. In view of the poor thermal conductivity of air (18), this study was designed to determine whether and to what extent these previously reported airway temperatures were underestimated by the indirect measurement of the exhaled air temperature.

We further hypothesize that the mucosal temperature in the tracheobronchial tree is non-uniformly distributed along the longitudinal direction since the inhaled air temperature increases progressively as it passes through the conducting airways toward the deeper regions of the lung. To answer these questions, we carried out this study using a miniature rapid-response temperature probe to measure directly the mucosal temperatures of trachea, major, lobar, and segmental bronchi in human subjects who underwent the bronchoscopy procedure for clinical diagnosis or treatment.

## Materials and Methods

The study protocol was approved by the Institutional Review Board (IRB) at the University of Kentucky.

### Subjects

After obtaining an informed consent, eight subjects (4 females and 4 males; average age 53.0 ± 3.84 years) who underwent the bronchoscopy procedure for diagnosis or treatment of non-infectious airway and/or lung disorders were recruited to participate in this study. All subjects had history of smoking although none were active smokers. While none of the subjects were febrile, two were on antibiotics at the time of bronchoscopy; however, cultures obtained from bronchoalveolar lavage were retained for 6–8 weeks and none were positive for bacterial, viral, mycobacterial, or fungal infections. The subject characteristics are presented in details in Table 1.

**Table 1:** Patient characteristics.

**Patient**	**Age**	**Sex**	**Weight (lb)**	**Height (in)**	**Race**	**Location of temperature measurements**
1	52	Female	182	64	White	UT, LT, RMB, Rt middle lobe, Rt middle lobe lateral segment
2	55	Male	246	71	White	UT, LT, LMB, Lt upper lobe, Lingula
3	46	Male	291	75	Black	UT, LT, LMB, Lt upper lobe, Lingula
4	58	Female	135	62	White	UT, LT, RMB, Rt middle lobe, Rt middle lobe lateral segment
5	62	Female	105	64	White	UT, LT, LMB, Lt upper lobe, Lingula, Lingular inferior segment
6	64	Male	150	77	White	UT, LT, RMB, Rt middle lobe, Rt middle lobe lateral segment
7	57	Female	118	63	White	UT, LT, LMB, Lt upper lobe, Lingula
8	30	Male	157	67	Hispanic	UT, LT, RMB, Rt upper lobe, Rt upper lobe anterior segment

*UT, upper trachea; LT, lower trachea; Rt, right; Lt, left; RMB, right main stem bronchus; LMB, left main stem bronchus*.

### Direct Measurement of Airway Mucosal Temperature

**Figure 1:**
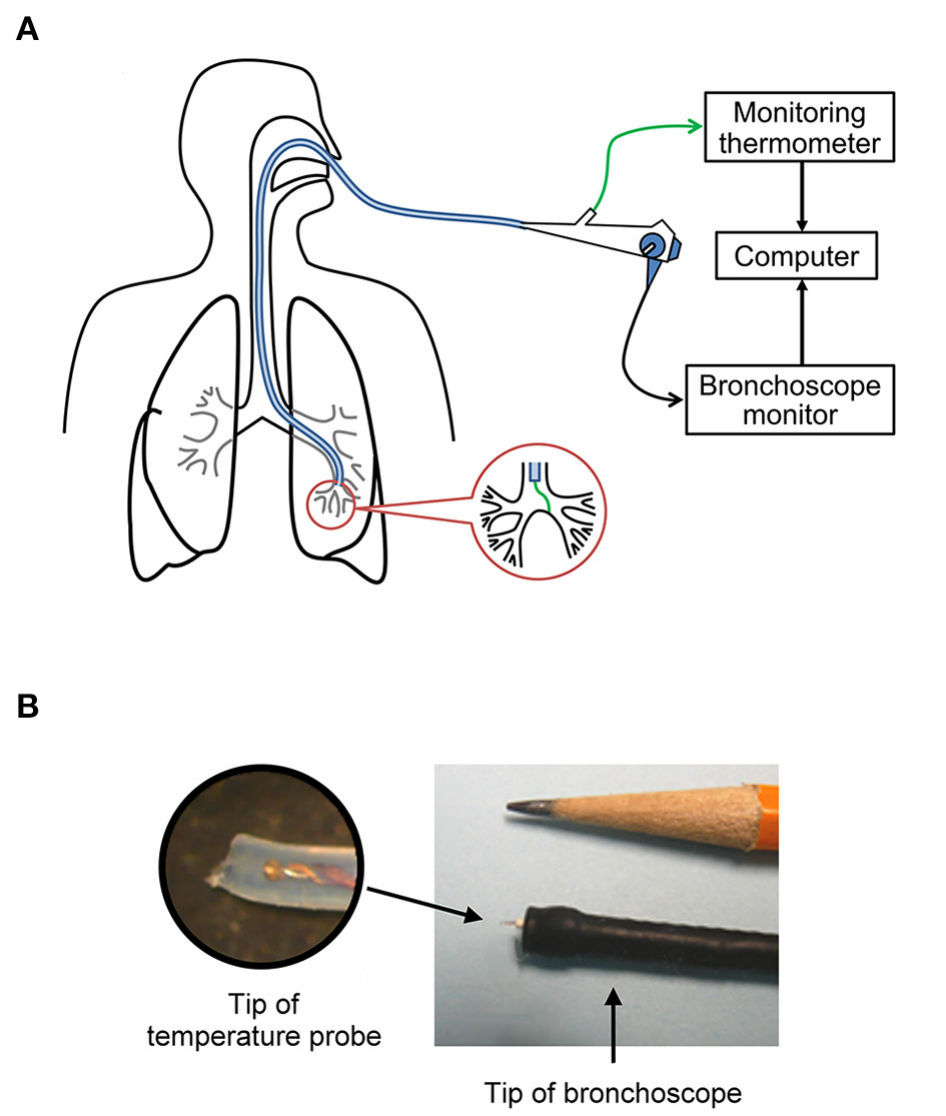
**(A**) Sketched diagram illustrating the direct measurement of airway mucosal tissue temperature via bronchoscopy. **(B)** The miniature temperature probe (outside diameter = 0.4 mm) was threaded through the instrument channel of the bronchoscope; the inset is an enlarged picture of the tip of the probe.

During the bronchoscopy procedure, subjects received premedication consisting of intravenous midazolam (0.5–2.0 mg) and fentanyl (25–100 μg) for a moderate level of sedation. Following nasopharyngeal anesthesia (topical lidocaine; 1%), a fiberoptic bronchoscope [Olympus model BF-H190; Waltham, MA, USA; outer diameter (OD) of the distal tip: 5.5 mm] was inserted via either nose (*n* = 4) or mouth (*n* = 4) and passed into the trachea. The direct measurement of airway mucosal temperature was then performed by passing a miniature rapid-response (time constant: 0.08 s) temperature probe (PhysiTemp IT-21; Clifton, NJ, USA) through the instrument channel of the bronchoscope (Figure 1A). The probe was gently pressed against the airway mucosa and held stationary for at least 5 s at each of the following locations of the respiratory tract: (1) upper trachea (extra-thoracic); (2) lower trachea (intra-thoracic); (3) main bronchus; (4) lobar bronchus; (5) segmental bronchus. The mucosal temperature at each location was then averaged for 5 s in each subject, and these measurements were made in all 8 subjects, except for the segmental bronchus (*n* = 5). The temperature probe has a soft and blunted tip made of medical grade of Teflon tubing (OD = 0.4 mm: Figure 1B), and its application for measuring temperature in human airways was evaluated by FDA for the investigational design exemption and approved as non-significant risk.

**Figure 2:**
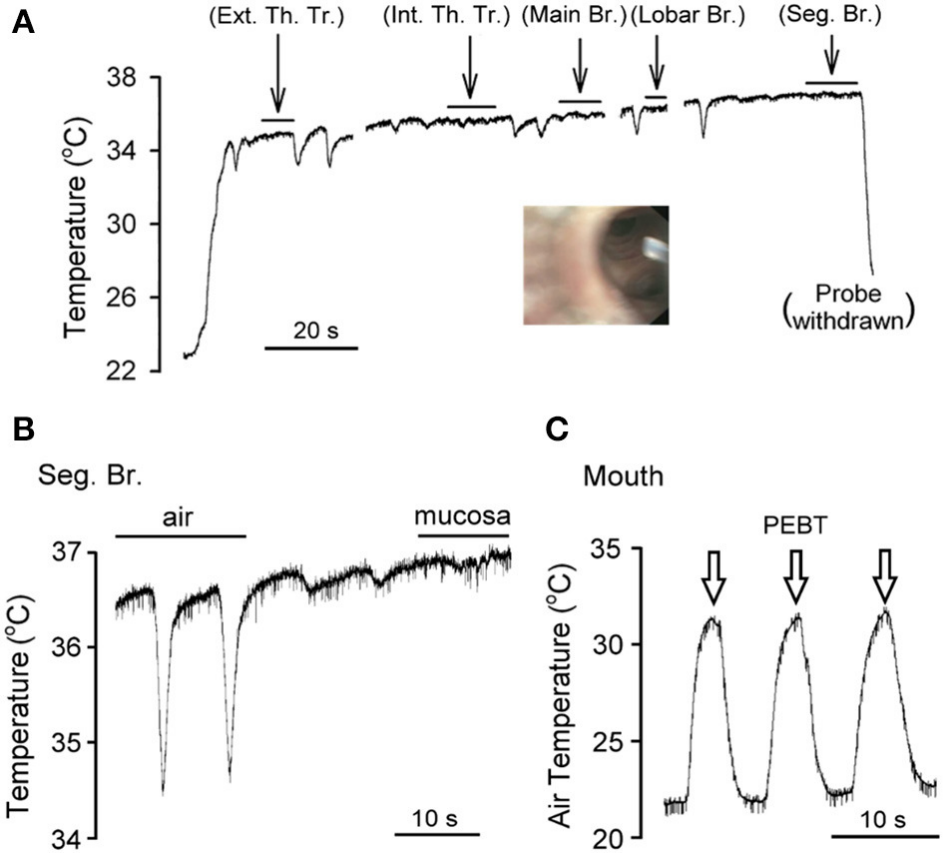
An experimental record illustrating the direct measurement of airway mucosal temperature in the right middle lobe, lateral segment of a subject (female, white, age 52) under light sedation. **(A)** As the tip of the bronchoscope was advanced continuously from trachea toward segmental bronchus, the temperature probe was applied to the airway mucosa and held stationary for the durations marked by the horizontal bars (>5 s in each location); when the probe was slowly advanced in the airway lumen (not in direct contact with the tissue), downward temperature spikes were generated by inspirations. Ext. Th. Tr., extrathoracic trachea; Int. Th. Tr., intrathoracic trachea; Br. bronchus; Seg. Br., segmental bronchus. The inset was a picture taken by the bronchoscope positioned at the right middle lobar br., showing the tip of temperature probe in the airway lumen facing the openings of two seg. br. **(B)** Experimental trace was enlarged to illustrate the difference between tissue and air temperature in the seg. br. of the same subject. **(C)** The peak (end-tidal) exhaled breath temperature (PEBT, marked by open arrows) was measured when the temperature probe was positioned in the mouth.

Both inhaled and exhaled air temperatures were recorded at the opening of the mouth using the same temperature probe during quiet breathing for at least three consecutive tidal breaths (e.g., Figure 2C) immediately before the bronchoscopy procedure, and the PEBT of the exhaled air was recorded, analyzed and averaged for 3 breaths in each subject.

The temperature signal obtained from the probe and monitoring thermometer (PhysiTemp model TH-8; Clifton, NJ, USA) was recorded continuously (Biopac model TSD160A; Goleta, CA, USA) and analyzed by a data acquisition system (Biocybernetics model TS-100; Taipei, Taiwan).

### Statistical Analysis

Data were analyzed with the one-way repeated-measures analysis of variance (ANOVA). When ANOVA showed a significant positive interaction, pair-wise comparisons were made with a *post-hoc* analysis (Fisher's least significant difference). A value of *P* < 0.05 was considered significant. Data are reported as means ± SEM.

## Results

The average oral temperature of the 8 subjects was 36.6 ± 0.1°C; room temperature was 21.8 ± 0.1°C. The PEBT of exhaled air, measured via the mouth or nose and averaged for three consecutive breaths in each subject, was 31.7 ± 0.8°C (*n* = 8; e.g., Figure 2C).

The bronchoscope tip was inserted through the nasal and oral passages equally (*n* = 4 each) in these eight subjects. The air temperature measured in the airway lumen by the temperature probe via the bronchoscope increased and decreased during expiratory and inspiratory phases, respectively, of each breath (e.g., Figure 2). This “tidal” change in air temperature coincided with the respiratory cycle. Although the amplitude of this phasic difference in air temperature appeared to dwindle toward more distal segments of the airways, it was clearly present even in the segmental bronchi (Figure 2B). In contrast, when the tissue temperature was measured directly by pressing the probe against the airway mucosa and held in a stationary position, it remained relatively stable, with distinctly smaller or almost undetectable phasic changes during respiratory cycles (e.g., under the horizontal bars in Figure 2A).

**Figure 3:**
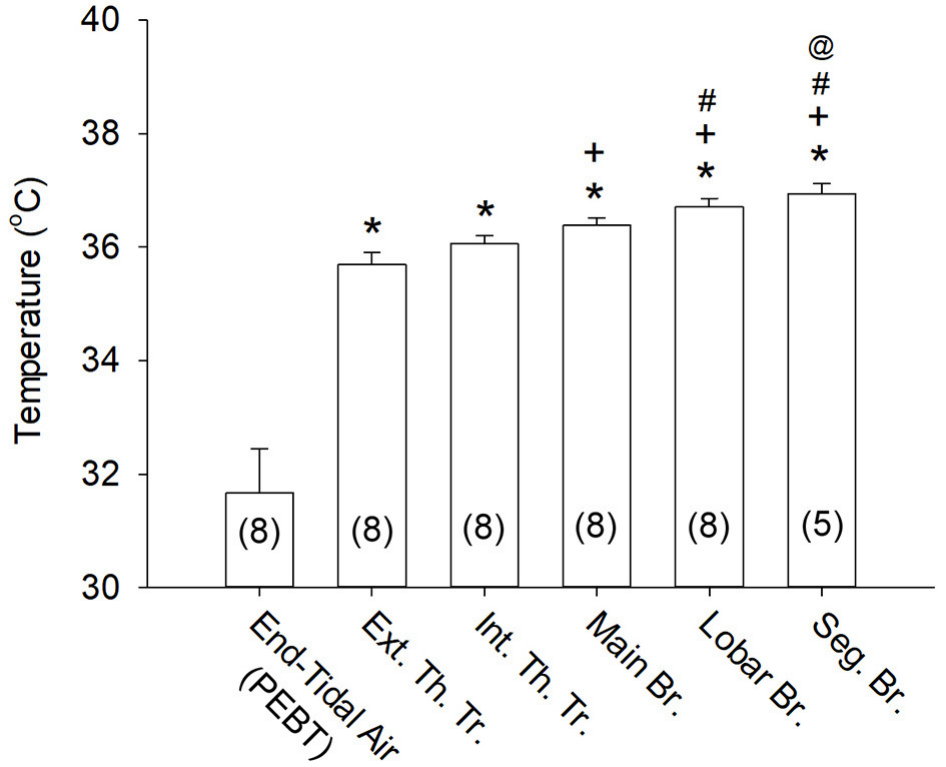
Airway mucosal temperatures measured at different locations of the respiratory tract. The mucosal temperature at each airway location was averaged over 5 s (see Figure 2A) in each subject; the end-tidal air temperature (PEBT) was measured in the mouth at the peak of exhaled breath (see an example in Figure 2C) and averaged for 3 consecutive breaths. Data were collected from 8 subjects, except at the segmental bronchus (*n* = 5). *P < 0.001, significantly different from the end-tidal air temperature (PEBT); ^+^*P* < 0.005, significantly different from the Ext. Th. Tr. mucosal temperature; ^#^*P* < 0.01, significantly different from the Int. Th. Tr. mucosal temperature; ^@^*P* < 0.01, significantly different from the Main Br. mucosal temperature. See legend of Figure 2 for descriptions of abbreviations.

Airway mucosal temperature (averaged for 5 s in each location) increased progressively from extra-thoracic trachea (35.7 ± 0.2°C, *n* = 8), intra-thoracic trachea (36.1 ± 0.2°C, *n* = 8) to more distal regions of the respiratory tract (Figures 2, 3). The mucosal temperature in the segmental bronchus (36.9 ± 0.2°C; *n* = 5) was not significantly different from that in the lobar bronchus (36.7 ± 0.1°C; *n* = 8, *P* > 0.05), but significantly higher than that in the main bronchus (36.4 ± 0.1°C; *n* = 8, *P* < 0.01); the latter was in turn higher than that in the extra-thoracic trachea (*n* = 8, *P* < 0.005). There was no significant difference in the mucosal temperatures between extra-thoracic and intra-thoracic trachea (Figure 3; *n* = 8, *P* > 0.1).

More importantly, the mucosal tissue temperatures measured in each of these airway segments was substantially higher than the PEBT measured in the exhaled air (averaged for 3 breaths) via the mouth or nose (31.7 ± 0.8°C; *n* = 8, *P* < 0.001). These observations clearly showed that the airway tissue temperature is significantly underestimated by the measurement of the PEBT (Figure 3). This difference was consistently detected even in the intrathoracic airways; for example, in the segmental bronchus the end-tidal exhaled air temperature was ~0.4°C lower than the mucosal tissue temperature (Figure 2B).

We separated the data obtained from these 8 subjects into two sub-groups based upon the route that the bronchoscope was inserted: nasal (*n* = 4) and oral (*n* = 4). We did not find consistently detectable difference in the mucosal temperature measured at any of the airway locations between these two subgroups.

## Discussion

A deviation of airway tissue temperature from normal level may reflect a pathophysiological condition of the airways (3, 4), and it can also lead to abnormal changes in airway functions (1, 2, 15, 17). However, the actual tissue temperature determined by a direct measurement in human airways was not yet known. Other approaches in attempt to measure the mucosal temperature in human respiratory tract using non-invasive methods such as infrared or thermal radiation thermometer and nuclear magnetic resonance imaging technology were either not feasible or unsuccessful due to the limited accessibility to the visceral locations and inability to obtain accurate and reliable data during respiratory movements. In this study, we have measured directly the mucosal temperature along the longitudinal direction of respiratory tract from trachea to segmental bronchus in awake human subjects.

Because the lung and airways are enclosed in the thoracic chamber (except the extra-thoracic tracheal segment), it seems logical to assume that their tissue temperatures are constantly maintained at the body core temperature. However, airway lumens are also continuously exposed to ambient air temperature during each inspiratory cycle, which can lower the temperature of airway mucosa. Indeed, our results showed that the airway mucosal temperature increased progressively from the extra-thoracic trachea (35.7 ± 0.2°C) toward more distal regions of the lung (segmental bronchus; 36.9 ± 0.2°C). These data are in general agreement with previous reports of the difference in air temperature along the respiratory tract (19, 20). This difference was not due solely to the higher (core) temperature within the thoracic chamber because there was also a significant difference between the mucosal temperature in the intra-thoracic trachea (36.1 ± 0.2°C) and that in the segmental bronchus (Figure 3). In addition, there was no significant difference in temperature between extra-thoracic and intra-thoracic tracheas (Figure 3). We speculate that this progressive decrease in airway mucosal temperature was, in part, related to the cooling effect generated by the inhaled ambient air on the airway mucosa. Because the total cross-sectional area of airway lumen increases continuously and progressively toward the more distal generations of the airways. Therefore, the linear velocity of the inhaled airflow will decease progressively along the same longitudinal direction of the tracheobronchial tree (21). As such, the cooling effect generated by inhaled air flow on the airway mucosa will decline toward the more peripheral airways. There are also other factors that may contribute to this difference in mucosal temperatures between trachea and intrathoracic airways; for example, a difference in the rates of heat transfer via conduction and convection between air and tissue at different regions of the tracheobronchial tree has been reported in a recent study when the PEBT was measured in the sequential fractions of the single-breath exhaled air in human subjects (20). In addition, differences in local blood flow (20) and mucosal tissue structures may also play a part in the difference of mucosal temperatures between these airway segments.

The average mucosal temperature in the segmental bronchus (36.9 ± 0.2°C) was not statistically different from the oral temperature measured in the same subjects (36.6 ± 0.1°C; *P* > 0.05). Since the oral temperature is known to be 0.5°C lower than the body core temperatures in human subjects at rest (22), it seems logical to assume that the tissue temperatures in the airways distal to the segmental bronchus are approximately the same as the core temperature. However, if an inflammatory reaction occurs in the lung, it is conceivable that the local tissue temperature in the inflamed region(s) may rise above the body core temperature (23, 24).

In a previous study using the whole-cell patch clamp electrophysiological recording technique, we have demonstrated that vagal sensory neurons innervating the airway mucosa exhibited exquisite sensitivity to temperature changes within the physiological range (8, 12); these isolated neurons displayed a distinct change in the neural activity in response to a change in temperature in the range similar to that occurred in the respiratory cycles (8). However, contrary to our prediction in the present study, despite the cyclic change of the air temperature in the airway lumen in phase with respiratory cycles, the mucosal temperature in the airways stayed at a relatively constant level and did not exhibit clear phasic changes during quiet breathing (Figure 2). This may be related to the poor thermal conductivity of air (18) and the short duration of each respiratory cycle, which did not allow the air temperature to reach equilibrium with that in the airway mucosa.

Most importantly, results obtained in this study clearly indicate that the end-tidal PEBT (31.7 ± 0.8°C) measured via the mouth or nose does not accurately represent the tissue temperature in the airway mucosa. This distinct difference between the air and mucosal temperatures was also evidently present even in the intrathoracic airways (e.g., segmental bronchus); an example is shown in Figure 2B. It is imperative to recognize this difference in temperature between airway tissue and exhaled air for the following reason. A recent study has reported that the end-expiratory air temperature plateau was 2.7°C higher in mild allergic asthmatic children than in healthy children, with no difference in the body temperature between the two groups (4). Furthermore, the difference in the end-expiratory air temperature was closely correlated with the indexes of allergic airway inflammation such as exhaled nitric oxide concentration as well as the sputum eosinophil percentage (4). Indeed, this observation is consistent with the fact that an increase in local tissue temperature is viewed as a hallmark feature of inflammatory reaction in various tissues and organs (23, 24). In addition, an earlier study also reported a faster rise of exhaled air temperature in adult asthmatics than matching controls (3). Taken together, these findings clearly revealed a close association between an increase in the PEBT and the degree of airway inflammation during asthma exacerbation, which suggested the potential application of the PEBT measurement in clinical diagnosis and its physiological implication (3, 4, 16). However, based upon our observations described in this study, we believe that the actual increase in the airway tissue temperature during asthma exacerbation is likely to be considerably higher than that estimated from the end-expiratory air temperature (3, 4).

An abnormal change in the airway mucosal temperature can also lead to the development of abnormal changes in airway functions. For example, breathing cold dry air induces bronchoconstriction in asthmatics, which is a well-documented pathophysiological condition known as cold air- or exercise-induced bronchoconstriction resulting from the injury of airway mucosa and the subsequent releases of various bronchoactive mediators such as leukotrienes and histamine (2). In addition, we have recently demonstrated that hyperventilation of warm humid air for 4 min induced a mild increase in the PEBT (Δ = 1.4–1.6°C) accompanied by an immediate bronchoconstriction and vigorous coughs in patients with mild allergic asthma (15) and allergic rhinitis (17), respectively. Based upon the finding of the present study, we suspected that the actual increase in airway mucosal temperature generated by hyperventilation of warm humid air might have been much higher. In that study, the bronchoconstriction was prevented by ipratropium in patients with asthma, suggesting an involvement of cholinergic reflex and an activation of temperature-sensitive sensory nerves innervating the airways (10, 15). Indeed, the TRP vanilloid type 1 (TRPV1) receptor, a biological temperature sensor (12), are abundantly expressed in the sensory nerves innervating the airway mucosa (8, 9, 25); and an over-expression of the TRPV1 receptor has been demonstrated in the airways of patients with chronic airway inflammatory diseases (7, 13, 26). Whether an activation of TRPV1 was responsible for the cholinergic reflex bronchoconstriction triggered by an increase in airway tissue temperature in asthmatic patients observed in our previous study remains to be determined. It should be noted, however, that a distinct link of high ambient (inhaled) air temperature to acute asthma exacerbation and airway dysfunction has been recently reported in an increasing number of epidemiological and environmental studies (27–30).

In this study, the bronchoscope tip was inserted through either nasal or oral passage in an equal number of subjects: four in each group. Theoretically, when the subject breathes through nose, the inspired air temperature is expected to be slightly higher (than that through mouth) when the air is inhaled into the trachea and bronchi, due to a more efficient warming and humidifying function of the nasal mucosa. Despite this expectation, we did not find a consistent or significant difference in the airway mucosal temperature between these two subgroups of subjects at any of the airway locations during quiet spontaneous breathing. However, we cannot rule out the possibility that the difference in airway mucosal temperature between these two different routes of breathing can be amplified by hyperventilation, such as during exercise (1, 18).

Certain perceived limitations of this study should be noted here. For example, because of the invasive nature of the bronchoscopy procedure, this approach cannot be applied as a routine procedure to measure the airway tissue temperature directly for clinical diagnosis. For the same reason, we did not recruit healthy volunteers to participate in this study. Instead, we recruited subjects who required bronchoscopy for treatment or diagnostic purpose, but were not febrile and did not have any active infectious or overtly systemic inflammatory diseases. In the cases presented with multiple pulmonary nodules, mass or lymphadenopathy, the conditions were localized and stable. Patients who presented with inflammation, chronic infection or malignancy in the lung or airways as revealed by the bronchoscopy were excluded. In addition, we excluded the subjects who had fever or signs of active systemic infection or inflammation at the time of screening or had a positive infection or active malignancy based on their final bronchoscopy results. Thus, we believe that the regions of the lungs where the airway temperatures were measured in this study were in relatively normal physiological conditions. Another potential limitation is that our study data were collected from only eight subjects. However, despite the relatively small sample size, the statistical analysis indicted a highly significant difference between data points, lending a strong support to our conclusion.

In conclusion, this study has clearly demonstrated that the airway mucosal tissue temperature was substantially underestimated by the measurement of PEBT air temperature. More importantly, the recent findings of a close association between an increased PEBT and airway tissue inflammation has revealed the potential of incorporating the PEBT measurement in the future clinical diagnosis of airway inflammation. In addition, considering the important role of airway temperature as a regulatory signal mediated through temperature sensors (e.g., TRP channels) expressed in the airway sensory nerves, the direct measurement of the airway mucosal temperature in this study has provided new information describing the physiological profile of this parameter along the human respiratory tract.

## Data Availability Statement

The original contributions presented in the study are included in the article/supplementary material, further inquiries can be directed to the corresponding author/s.

## Ethics Statement

The studies involving human participants were reviewed and approved by The Institutional Review Board (IRB) at the University of Kentucky. The patients/participants provided their written informed consent to participate in this study.

## Author Contributions

MK and L-YL: design of study. MK, R-LL, AM, SP, and L-YL: execution of experiment. MK, R-LL, A-HL, and L-YL: data analysis. MK, R-LL, AM, SP, A-HL, and L-YL: data interpretation and drafting, reading, and editing of the manuscript. All authors contributed to the article and approved the submitted version.

## Conflict of Interest

The authors declare that the research was conducted in the absence of any commercial or financial relationships that could be construed as a potential conflict of interest.

## Publisher's Note

All claims expressed in this article are solely those of the authors and do not necessarily represent those of their affiliated organizations, or those of the publisher, the editors and the reviewers. Any product that may be evaluated in this article, or claim that may be made by its manufacturer, is not guaranteed or endorsed by the publisher.
